# Antibiotic Susceptibilities of *Enterococcus* Species Isolated from Hospital and Domestic Wastewater Effluents in Alice, Eastern Cape Province of South Africa

**DOI:** 10.3390/ijerph120404231

**Published:** 2015-04-16

**Authors:** Benson Chuks Iweriebor, Sisipho Gaqavu, Larry Chikwelu Obi, Uchechukwu U. Nwodo, Anthony I. Okoh

**Affiliations:** 1 SA-MRC Microbial Water Quality Monitoring Centre, University of Fort Hare,1 King Williams Town Road, Alice 5700, South Africa; E-Mail: gaqavus@gmail.com; 2 Applied and Environmental Microbiology Research Group, Department of Biochemistry and Microbiology, University of Fort Hare, 1 King Williams Town Road, Alice 5700, South Africa; E-Mails: unowdo@ufh.ac.za (U.U.N.); aokoh@ufh.ac.za (A.I.O.); 3 Academic and Research Division, University of Fort Hare, King Williams Road, Alice 5700 Eastern Cape, South Africa; E-Mail: lobi@ufh.ac.za

**Keywords:** hospital, wastewaters, antibiotic resistance, virulence, enterococcus species

## Abstract

Background: Antimicrobial resistance in microorganisms are on the increase worldwide and are responsible for substantial cases of therapeutic failures. Resistance of species of *Enterococcus* to antibiotics is linked to their ability to acquire and disseminate antimicrobial resistance determinants in nature, and wastewater treatment plants (WWTPs) are considered to be one of the main reservoirs of such antibiotic resistant bacteria. We therefore determined the antimicrobial resistance and virulence profiles of some common *Enterococcus* spp that are known to be associated with human infections that were recovered from hospital wastewater and final effluent of the receiving wastewater treatment plant in Alice, Eastern Cape. Methods: Wastewater samples were simultaneously collected from two sites (Victoria hospital and final effluents of a municipal WWTP) in Alice at about one to two weeks interval during the months of July and August 2014. Samples were screened for the isolation of enterococci using standard microbiological methods. The isolates were profiled molecularly after targeted generic identification and speciation for the presence of virulence and antibiotic resistance genes. Results: Out of 66 presumptive isolates, 62 were confirmed to belong to the *Enterococcus genusof* which 30 were identified to be *E. faecalis* and 15 *E. durans*. The remaining isolates were not identified by the primers used in the screening procedure. Out of the six virulence genes that were targeted only three of them; *ace, efaA*, and *gelE* were detected. There was a very high phenotypic multiple resistance among the isolates and these were confirmed by genetic analyses. Conclusions: Analyses of the results obtained indicated that hospital wastewater may be one of the sources of antibiotic resistant bacteria to the receiving WWTP. Also, findings revealed that the final effluent discharged into the environment was contaminated with multi-resistant enterococci species thus posing a health hazard to the receiving aquatic environment as these could eventually be transmitted to humans and animals that are exposed to it.

## 1. Introduction

Hospitals and clinics are major reservoirs for large numbers of pathogenic bacteria comprised of resident and community introduced strains [1]. High usage of antibiotics to treat infections in patients serves as a selective pressure for resistance development and there are concerns with transmission and their long term survival in the environment [2]. Dissemination of antibiotic resistant bacteria (ARB) from hospitals can occur via various routes such as hospital wastewater, discharged patients and health care workers [2,3]. Antibiotics in wastewater can arise from excretion in urine and faces, direct disposal of expired drugs, and accidental spilling; these events could serve as additional selective pressure on bacteria while in wastewater. An elevated level of antibiotics and other pharmaceuticals in the environments are considered favorable for the selection of antibiotic resistance and most probably important hotspots for horizontal gene transfer (HGT) of resistance genes, and therefore conducive sites for resistance evolution [4,5,6]. The possible persistence and further dissemination of ARB in natural aquatic environments could ultimately lead to an increase in the pool of antimicrobial resistance determinants. The transfer of resistance into current and emerging pathogens are major concerns that are being entertained with regards to the continuous introduction of ARB and their resistance genes into the environment.

*Enterococcus* spp. are Gram-positive, non-spore-forming organisms that belong to a group of organisms known as lactic acid bacteria with some species producing a protein called bacteriocin. They are commensal members of the normal intestinal flora of humans and animals and female genitourinary tract without causing any infection; enterococciare commonly found in humans and animal faces, and faecalenterococci e.g., *E. faecalis*, *E. faecium*, *E. durans* and *E. hirae* are used as indicators of sewage contamination [7]. They also indicate possible presence of disease causing pathogens that inhabit the gastrointestinal tracts of human and animals [8]. As indicators of the hygienic quality of water, *Enterococcus* spp. are considered to be more specific than the faecalcoli forms as a whole [9].

Even though enterococci are non-pathogenic under normal circumstances, they can become opportunistic pathogens once the commensally relationship with the host is disrupted [10]. The genus *Enterococcus* has more than 40 species. The most abundant species in the human intestine and most commonly isolated from enterococcal infection include *E. faecalis* and *E. faecium.* Other species that have also been detected include; *E. avium, E. casseliflavus, Enterococcus durans, E. sgallinarum* and *E. raffinosu* [11].

Enterococci may have different genes that directly or indirectly contribute to virulence [12]. Genes encoding virulence factors in enterococci include *asa*1, *efa*A, *ace, esp, cyl*A, *gel*E and *hyl*A [13]. The *asa*1 gene, a plasmid gene, encodes for an aggregation substance (AS) which is a protein that mediates binding to the host epithelium and this protein also facilitates plasmid exchange by mediating bacterial aggregation during conjugation.

The *efa*A gene is associated with evasion of the immune response. The *esp* gene encodes for the enterococcus surface protein (ESP), which participates in biofilm formation and is associated with colonization and persistence in the urinary tract. The *ace* gene encodes for a protein “adhesion of collagen” which mediates the association of bacteria to matrix protein of the host cell. The *hyl*A gene encodes for hyaluronidase which plays a role in increasing bacterial invasion. The *cylA* gene encodes for cytolysis which has the ability to lyse a range of cells both prokaryotic and eukaryotic. The *gelE* gene encodes for Zn-metalloendopeptidase, which has the ability to hydrolyzegelatine, casein, and hemoglobin [14].

One major attribute of the genus *Enterococcus* is its propensity to acquire and disseminate antimicrobial resistance determinants. Enterococci have become resistant to a wide range of antibiotics and resistance to penicillin, cephalosporins, amino glycosides, and clindamycin are due to the various intrinsic traits they express [15] while resistance to chloramphenicol, erythromycin, tetracycline, fluoroquinolones and vancomycin are examples of acquired resistance [15]. Nearly 30 years after vancomycin was medically introduced, vancomycin-resistant enterococci were reported and the trend has increased since then [16,17,18,19]. In the late 1980s, the first clinical isolates of vancomycin-resistant strains of pathogenic *E. faecalis and E. faecium* appeared. That vancomycin resistant bacteria have the ability to transfer the vancomycin resistance determinant to other bacteria poses threats to human health. There are many documented phenotypes of vancomycin resistance which includes *van*A, *van*B, *van*C, *van*D, and *vanE* [20].Resistance phenotypes *vanA* and *van*B were described mainly in *E. faecalis* and *E. faecium*. Resistance phenotype *van*C was described in *E. casseliflavus* and *E. Gallinarum* [19].

This study was aimed at determining the antimicrobial resistance patterns of *Enterococcus* spp. recovered from both hospital and municipal wastewaters in Alice, Eastern Cape Province, South Africa as a continuation of our larger study on the reservoirs of antibiotic resistance in the environment.

## 2. Materials and Methods

### 2.1. Sample Collection

The Victoria hospital and the Fort Hare wastewater treatment plant are both situated in Alice. Alice is a university and an administrative headquarters of Noncore local Municipality, in the Amatole District of the Eastern Cape in South Africa. The Fort Hare WWTP receives Victoria hospital wastewater, domestic sewages and runoff water within Alice town. The wastewater originating from the Victoria hospital does not have any form of pretreatment before being discharged into the main drain that eventually goes to the Fort Hare wastewater treatment plant Wastewater samples were collected from two sites in Alice (discharged wastewater from Victoria hospital and the final effluent of the receiving wastewater treatment plant simultaneously). Sampling was carried out at regular intervals between the months of June and August, 2014. Two hundred milliliters (200 mL) of hospital wastewater and final effluent were collected per site. The wastewater was collected at the discharge point into the main storm drain while the final effluent was taken as it was being released into the receiving water body. A total of 64 samples were collected altogether; 32 samples from each sampling site during the sampling period. Samples were collected in sterilized 350 mL bottles, kept in ice boxes and transported to the laboratory for microbiological analysis. 

#### 2.1.1. Isolation, Identification, DNA Extraction and Molecular Confirmation and Speciation of *Enterococcus* spp.

Bacteriological analyses were performed by inoculating tubes containing 9.5 ml Tryptic Soy Broth (TSB) with 0.5 mL of the samples and incubated for 18 h at 37 °C. Tubes showing bacterial growths from each site were streaked onto selective agar plates (Bile Aesculin Azide Agar).The plates were incubated at 37 °C for 24 h. An isolated colony per plate with appropriate colonial characteristics in the differential medium was selected as presumptively positive [21].The ability to hydrolyze esculin in the presence of bile is a characteristic of enterococci which results in the production of esculent in that reacts with ferric citrate to form a dark brown or black colony. These black colonies were regarded as presumptive isolates and one colony per plate was further inoculated into TSB, and incubated at 37 °C for 24 h from which glycerol stock was prepared for future use.

The boiling method was used for DNA extraction upon resuscitation from the glycerol stock using TSB after overnight incubation at 37 °C. The bacterial suspension was centrifuged at 10,000 rpm for 10 min and supernatant was discarded and pellet washed with 200 µL saline and further centrifuged at 10,000 rpm for 10 min as previously described by Bai [22]. The supernatant was discarded and 150 µL of lysis buffer was added and cells were lysed in a heating block at 100 °C for 10 min. Lysed cells were centrifuged at 10,000 rpm for 10 minutes and the supernatant was used as DNA template.

A procedure previously described by Ke [23] was used for identification of the *Enterococcus* spp. Identification of enterococci was based on the detection of the genus-specific *tuf*-gene (product size 112 bp). The positive control used was *E. feacalis* ATCC 19433. The reaction mixture (25 µL) contained: 5 µL of the DNA template, 12 µL of Master Mix, 1 µL of each primer- Ent1 and Ent2, and 5 µL of nuclease free water. Sequences of applied primers are: Ent1 5’-TACTGACAAACCATTCAT GAT G-3’ and Ent2 5’-AACTTCGTCACCAACGCGAAC-3’. The PCR cycling conditions consisted of the initial denaturation 94 °C/3 min, amplification—30 cycles (94 °C/30 s, 53 °C/45 s, 72 °C/60 s), final extension 72 °C/7 min. Amplification was verified by gel electrophoresis in 2% agarose stained with ethidium bromide and visualized with UV transluminator (ALLIANCE 4.7, Cambridge, United Kingdom,) and photographed.

A polymerase chain reaction (PCR) was performed for *Enterococcus* species identification as previously described by Jackson [11] in a singleplex PCR with primer pairs shown in Table 1. Amplification of species-specific genes was performed for the identification of these following species: *E. faecalis, E. faecium, E. hirae, E. durans*, and *E. casseliflavus*. The Dream Taq PCR Master Mix (2×) consisting of 4 mM MgCl_2_, 0.4 m Mdeoxynucleoside triphosphate mix and Taq polymerase enzyme (Thermo Scientific, Pittsburgh, PA, USA) and 10 p Mol of each primer pair was added to the reaction mixture in a PCR tube. PCRs were performed in a final volume of 25 µL consisting of 20 µL of master mix and 5 µL of DNA template. Following an initial denaturation at 95 °C for 4 min, products were amplified in 30 cycles of denaturation at 95 °C for 30 s, annealing at 52°C (*E. faecalis, E. durans* and *E. casseliflavus*) or 48 °C (for *E. faecium and E. hirae*) for 1 min, and elongation at 72 °C for 1 min followed by a final extension at 72 °C for 7 min. Five microliters of product was electrophoresed on a 2% Tris-borate-EDTA agarose gel containing 2 µg of ethidium bromide/ml to verify amplification of the targeted genes at 110 V for 45 min. A DNA molecular weight marker of 100 bp was used as the standard and photographed under UV light transilluminator (ALLIANCE 4.7) Molecular Imager Gel Doc.

**Table 1:** PCR primers, products, and reference strains.

Strain	Primer	Sequence (5’–3’)	Product Size(bp)	Ref.
*E.faecalis* ATCC 19433	FL1 FL2	ACTTATGTGACTAACTTAACC TAATGGTGAATCTTGGTTTGG	360	[11]
*E.durans* ATCC 19432	DU1 DU2	CCTACTGATATTAAGACAGCG TAATCCTAAGATAGGTGTTTG	295	[11]
*E.casseliflavus* ATCC 25788	CA1 CA2	TCCTGAATTAGGTGAAAAAAC GCTAGTTTACCGTCTTTAACG	288	[11]
*E.faecium* ATCC19434	FM1FM2	GAAAAAACAATAGAAGAATTAT TGCTTTTTTGAATTCTTCTTTA	215	[11]
*E.hirae* ATCC 8043	HI1 HI2	CTTTCTGATATGGATGCTGTC TAAATTCTTCCTTAAATGTTG	187	[11]

#### 2.1.2. Detection of Virulence Genes

Two multiplex PCRs were used to screen for six virulence genes as previously described by [24,25] with little modification making use of the primer pairs as shown in Table 2. The six virulence genes screened among all the confirmed *Enterococcus* isolates were *ace, efa*A, *gel*E, *esp, cyl and hyl.* Multiplex group 1 was *ace* and *gel*E and multiplex group 2 were *efa*A, *esp, cyl* and *hyl*.

PCR cycling conditions used for multiplex group 1 was as follow: initial denaturation at 94 °C for3 min; followed by 35 cycles of amplification (93 °C/1 min, 50 °C/1 min, 73 °C/1 min) and final extension at 72 °C/10. Similar conditions were used for multiplex group 2 except that the annealing temperature was 56.5 °C/1 min. The products were resolved in 2% gel electrophoresis stained with ethidium bromide and visualized with UV transluminator (ALLIANCE 4.7) and photographed.

**Table 2:** Primers used to screen for virulence genes.

Gene and Primers	Sequence	Product Size (bp)	Ref.
*ace*		320	[24]
ACE1	5’-AAAGTAGAATTAGATCCACAC-3’		
aceACE1ACE2	5’-TCTATCACATTCGGTTGCG-3’		
*gelE*		402	[24]
gelE1	5’-AGTTCATGTCTATTTTCTTCAC-3’		
gelE2	5’-CTTCATTATTTACACGTTTG-3’		
*efaA*		499	[24]
efaA1	5’-CGTGAGAAAGAAATGGAGGA-3’		
efaA2	5’-CTACTAACACGTCACGAATG-3’		
*esp*		913	[25]
ESP46	5’-TTACCAAGATGGTTCTGTAGGCAC-3’		
ESP47	5’-CCAAGTATACTTAGCATCTTTTGG-3’		
*hyl*		276	[25]
HYL n1	5’-ACAGAAGAGCTGCAGGAAATG-3’		
HYL n2	5’-GACTGACGTCCAAGTTTCCAA-3’		
*cylA*		688	[25]
CYT I	5’-ACTCGGGGATTGATAGGC-3’		
CYT II	5’-GCTGCTAAAGCTGCGCTT-3’		

### 2.2. Antimicrobial Susceptibility Testing

The Kirby-Bauer disk diffusion method was used as described by [26,27] to evaluate the antimicrobial susceptibility profiles of the isolates using standardized single disk on Muller-Hinton agar (MHA). Antibiotics that are more frequently used and prescribed in Victoria hospital were selected for testing which are erythromycin (15 µg), imipenem (10 µg), tetracycline (30 µg), cefotaxine (30 µg), gentamicin (10 µg), clindamycin (2 µg), kanamycin (30 µg), vancomycin (30 µg), ciprofloxacin (5 µg) and penicillin 10 units.

#### PCR Detection of *van*A, *van*B, *van*C1, *van*C2*/3* and *erm*(B) Genes

The genes encoding vancomycin resistance were investigated among the isolates using the previously extracted genomic DNA and PCRs were performed in a BioRad Thermal Cycler (Foster, CA, USA). The oligonucleotide primers for PCR amplifications were synthesised by Inqaba Biotech (Pretoria, South Africa). Primer sequences for *van*A, *van*B, *van*C1, *van*C2/3 genes were those previously described by Nam [28]. The list of the specific primers used in the study and their amplification products are shown in Table 3. The reactions were performed as singleplex in a total volume of 25 µL, using 5 µL of cell lysate as DNA template, 10 pmol of each of the forward and reverse primers, 12 µL of Dream Taq master PCR mix (Thermo Scientific, Pittsburgh, PA, USA) and 6µL of PCR water grade. Cycling conditions were as follows: a first denaturation step of 94 °C for 3 min, 35 cycles of denaturation at 94 °C for 1 min, annealing at 56.5 °C (*van*B, C1, and C2/3) and 55 °C for *erm* (B) for 1 min, extension at 72 °C for 1 min, followed by an elongation step at 72 °C for 10 min. The PCR products were analyzed in 2% agarosegel stained with ethidium bromide, electrophoresed at 110 V for 45 min, visualized under UV transilluminator (ALLIANCE 4.7) and photographed.

**Table 3:** Oligonucleotide primers used to identify resistance genes.

Gene(s)	Product Size (bp)	Primer Name Oligonucleotide Sequences (5’ to 3’)	Ref.
*van*A	314	AF-GCGCGGTCCACTTGTAGATA	[29]
		AR-TGAGCAACCCCCAAACAGTA	
*van*B	220	BF-AGACATTCCGGTCGAGGAAC	[29]
		BR-GCTGTCAATTAGTGCGGGAA	
*van*C-1	402	C1F-ATCCAAGCTATTGACCCGCT	[29]
		C1R-TGTGGCAGGATCGTTTTCAT	
*van*C-2/3	582	C2F-CTAGCGCAATCGAAGCACTC	[29]
*erm*(B)	320	C2R-GTAGGAGCACTGCGGAACAA	[30]
		BN1-CGAGTGAAAAAGTACTCAACCA	
		BN2-CGGTGAATATCCAAGGTACG	

## 3. Results

### 3.1. Identification of Enterococcus to Species Level

**Figure 1:**
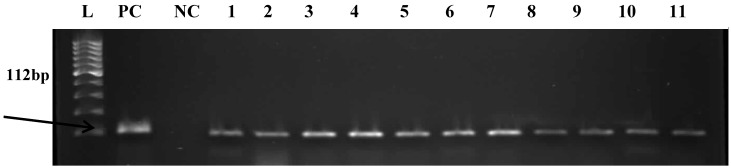
Gel imagerepresenting molecular confirmation of *tuf*-gene (112bp) of *Enterococcus* from hospital wastewater and final effluent of WWT P.L: ladder, PC: positive control *E. faecalis* ATCC 19433; NC: negative controlwas PCR mix without DNA; lanes 1–11 are confirmed enterococci isolates.

**Figure 2:**
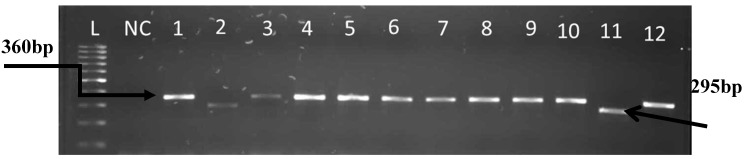
Gel imagerepresenting molecular confirmation of *Enterococcus* species *E. faecalis* (360bp), *E. durans* (295bp) from hospital wastewater and final effluent of WWTP. L: 100bp ladder (Gene Ruler); NC: negative control was PCR mix without DNA; lane 1 *E. feacalis* ATCC 19433; and lanes 2–12 are delineated isolates.

Out of 66 presumptive samples, 62 (93.93%) were confirmed to belong to the *Enterococcus* genus and made up of 33 from hospital wastewater and 29 from final effluents of the municipal WWTP. Some of the results for the presumptive identification and molecular confirmation of *Enterococcus* isolates are shown in the gel electrophoresis image in Figure 1. The 62 confirmed enterococci were further analyzed for species identification. The following species were targeted; *E. faecalis, E. durans, E. casseliflavus, E. faecium,* and *E. hirae* because of their frequent occurrence in human infections as reported in literatures. A total of 30 isolates (19 from hospital wastewater and 11 from the final effluent of the WWTP) were identified to be *E. faecalis* and 15 isolates (five from hospital wastewater and 10 from final effluent of WWTP) were identified to be *E. durans*. The remaining 17 isolates could not be delineated by the primes used in species identification because they were outside the species that were screened for. Some of the results for *Enterococcus* speciation are shown in the gel electrophoresis image in Figure 2.

### 3.2. Identification of Virulent Genes

**Figure 3:**
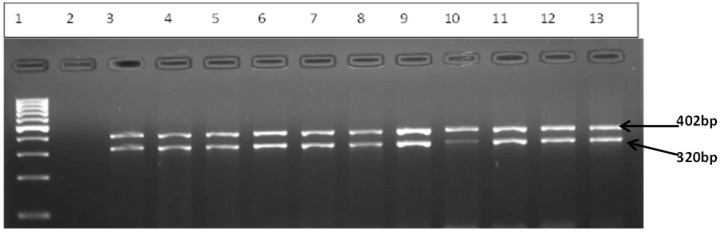
Gel image representing molecular identification of virulent genes *ace* (320bp) and *gel*E (402) from hospital wastewater and final effluent of WWTP. Lane 1 is 100bp ladder (Gene Ruler), lane 2 is negative control, lane 3–12: samples/isolates.

The DNA extract from the 62 confirmed enterococci isolates were further analyzed for virulence gene identification. Virulence genes that were targeted include *ace, efa*A, *gel*E, *esp, cyl* and *hyl*A. A total of 19 isolates that were positive for the *efa*A gene (15 from hospital wastewater and four from final effluent of WWTP); 15 isolates were positive for the *ace* gene (two from hospital wastewater and 13 from final effluent of WWTP), and three isolates were positive for the *gel*E gene (from final effluent). The rest of the isolates did not harbor any virulence genes. Some of the results for the enterococcus virulent genes obtained from this study are shown in the gel electrophoresis image in Figure 3.

### 3.3. Antimicrobial Susceptibility Profile of Confirmed Enterococcus Isolates from Hospital Wastewater

**Figure 4:**
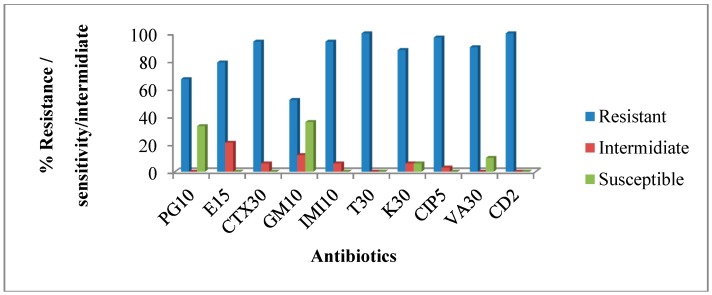
The percentage of Antimicrobial resistance profiles of isolates from hospital wastewater. PG 10= Penicillin10 µg; E15 = Erythromycin 15 µg; CTX30 = Cefotaxime 30 µg; GM10 = Gentamicin 10 µg; IMI10 = Imipenem 10 µg; T30 = tetracycline 30 µg; K30 = kanamycin 30 µg; CIP5 = ciprofloxacin 5 µg; VA30= vancomycin 30 µg; CD2 = clindamycin 2 µg.

Susceptibilities of the isolates from hospital wastewater were tested against ten different antibiotics and the results indicate high antibiotic resistance among the isolates. Approximately 67% to 100% of the isolates were resistant to penicillin, erythromycin, cefotaxime, gentamicin, imipenem, tetracycline, kanamycin, ciprofloxacin, vancomycin, and clindamycin (Figure 4). The observed phenotypic resistance patterns among the hospital wastewater isolates are shown in Table 4.

**Table 4:** Prevalence of phenotypic resistance patterns of isolates from hospital wastewater.

No. of Isolates	Phenotypic Multiple Resistance Patterns
16	PG/E/CTX/GM/IMI/T/K/CIP/VA/CD
6	PG/E/CTX /IMI/T/K/CIP/VA/CD
7	E/CTX /IMI/T/K/CIP/VA/CD
4	E/CTX/GM/IMI/T/K/CIP/CD

PG = Penicillin; E= Erythromycin; CTX = Cefotaxime; GM = Gentamicin; IMI = Imipenem; T= tetracycline; K = kanamycin; CIP = ciprofloxacin; VA = vancomycin; CD = clindamycin.

### 3.4. Antimicrobial Susceptibility Profile of Confirmed Enterococcus Isolates from the Final Effluent of Wastewater Treatment Plant.

**Figure 5:**
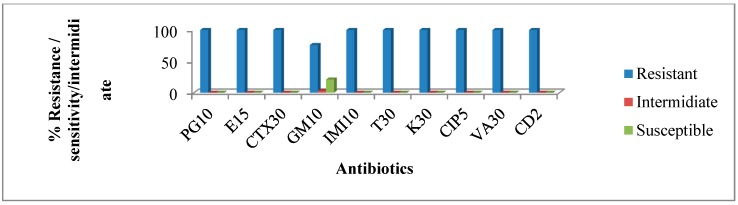
Antimicrobial resistance profiles of isolates from final effluent of WWTP. As seen in the graph, all the isolates were resistant to all the antibiotics with the exception of GM where resistance was 76%.

Results of antimicrobial resistance/susceptibility profiles of isolates from final effluent of WWTP showed that all the isolates were resistant against the entire antibiotic tested against them with the exception of gentamycin where 76% of the isolates were resistant (Figure 5) while the phenotypic resistance patterns are shown in Table 5.

**Table 5:** Phenotypic resistance patterns of the isolates from hospital wastewater.

Total No. of Isolates	Resistance Pattern of The Isolates
23	PG/E/CTX/GM/IMI/T/K/CIP/VA/CD
6	E/CTX /IMI/T/K/CIP/VA/CD

PG = Penicillin, E = Erythromycin, CTX = Cefotaxime, GM = Gentamicin, IMI = Imipenem, T = tetracycline, K = kanamycin, CIP = ciprofloxacin, VA = vancomycin, CD = clindamycin.

### 3.5. Detection of Antimicrobials Resistance Genes *from Confirmed* Enterococcus *Isolates*

Among the genes investigated for vancomycin resistance, *van*A was not detected while there was preponderance of *van*B, *van*C1 and *van*C2/3 genes. All the isolates that were phenotypically resistant to vancomycin were positive for *van*B, *van*C1 *van*C2/3 and 40 for *erm*(B) genes as shown in Table 6.

**Table 6:** Frequency of resistance genes to macrolideand glycopeptide detected among the isolates.

Antibiotic	Class	Resistance Gene	No. of Strain
Erythromycin	Macrolide	*erm*(B)	40 (88.8%)
Vancomycin	Glycopeptide	*van*B, *van*C1, *van* C2/3	42 (93.3%)

## 4. Discussion

Water pollution is one of the key environmental problems facing South Africa. This pollution arises from many sources which include municipal effluents. Surveys and technical papers have reported that since 2004, 70% of municipality waste treatment facilities in South Africa face collapse due to lack of proper maintenance and expansion [30]. The failures of wastewater treatment plants to efficiently remove contaminants result in the production of poor quality effluents that are capable of causing the degradation of the receiving water bodies, such as lakes, rivers, streams and dams.

In this study, *Enterococcus* spp. were isolated and identified in both the hospital wastewater and final effluent of the wastewater treatment plant. Presence of *Enterococcus* spp. in the final effluent shows that the Fort Hare wastewater treatment plant may not be quite efficient with regards to removing bacteria in the final effluent that is discharged to receiving water bodies. Since enterococci are also used as indicators of sewage contamination, its presence in the Fort Hare WWTP final effluent indicated possible presence of pathogens like bacteria, viruses, protozoans and helminthes [8]. This study shows the inefficiency of the chlorination process in killing microorganisms in the final effluent discharged into the environment. The discharged final effluents could have harmful effects on the receiving water body [30].

The confirmed enterococci from both hospital wastewater and final effluent of WWTP were further analyzed for species identification and 48.39% were *E. faecalis* (30% from hospital wastewater and 18% from final effluent) and 24% were identified as *E. durans* (8% from hospital wastewater and 16% from final effluent), while the rest were unidentified. *E. faecalis* has become an agent that is commonly associated with nosocomial infections, urinary tract infections, endocarditis, bacteremia, neonatal infections, central nervous system (CNS) infections, and abdominal and pelvic infections [31]. *E. durans* has been isolated from patients with enterococcal infection [32].Bacterial species in general and *E. faecalis* specifically, can activate several survival strategies including starvation and the viable but non culturable (VBNC) state [33,34] thus conserving their viability. Since enterococcal species may activate two different survival strategies, namely starvation and the VBNC state, depending on the specific environmental condition [35], this finding indicates that the final effluent discharged from the Fort Hare WWTP harboring *Enterococci* species is capable of causing infections in humans and animals that may come in contact with the discharged effluent.

Even though *Enterococcus* spp. are commensal in the guts of animals including humans, they are known to have virulence properties which could enable them to establish infections in certain categories of people whose immune systems have been compromised such as in elderly persons, children, pregnant women and long-term hospitalized patients. A high prevalence of genotypic virulence markers (*gel*E, *ace* and *efa*A) was detected in the isolated species, thus corroborating results of previous reports [24,36,37]. On the other hand, the following genes: -*hyl*A, *cyl*A and *esp* were absent in all species analyzed. The presence of *efa*A gene as previously reported by Abriouel [38], suggests that this gene is important for persistence of enterococci in environments other than human tissues. Virulence factors like collagen-binding protein (*ace*), enterococcal surface proteins (*esp*), and *E. faecalis* endocarditic antigen (*efa*A) plays a significant role in enterococcal adhesion to collagen and the extracellular matrix [39]. *E. faecalis* endocarditic antigen (*efa*A) was first identified from the antiserum of a patient with *E. faecalis* endocarditic [40]. The amino acid sequence of the associated protein *efa*A revealed 55%–60% homology to a group of streptococci proteins known as adhesions [40], hence it could be vital for attachment to surfaces and therefore aids in the organism’s persistence in the environment.

To determine antibiotic sensitivity of the isolates, we tested the isolates against a panel of antimicrobial agents most of which were drugs commonly prescribed at the hospital that generated the wastewater under investigation. The results are alarming as almost all the isolates were resistant to glycopeptides (vancomycin) and macrolide (erythromycin). For hospital wastewater, 91% were resistant to vancomycin and isolates from the final effluent were 100% resistant. Several studies have documented a high incidence of antibiotic resistance in such wastewaters all over the world [4,41,42]. 

Out of several different genes mediating vancomycin resistance, *van*A, *van*B, *van*C1, and *van*C2/3 resistance genes were targeted for identification as these gene clusters can be acquired and often are transferable [20]. Genetic profiling of the resistance genes was successful for *erm*B, *van*B, *van*C1 and *van*C2/3 genes. Genetic determinants for vancomycin resistance were homogeneously detected in most of the isolates the source notwithstanding. Similarly, *erm*B was also present in more than 80% of the isolates that were phenotypically resistant to erythromycin and this is in agreement with previous reports on erythromycin resistance in *Enterococci* recovered from wastewaters [2,43,44,45,46]. These results showed that the WWTP could act as a reservoir of antibiotic resistant bacteria as well as resistant determinants. The results of this study arein agreement with a similar study that has reported high rates of antibiotic resistant bacteria in the wastewater environment [47]. Also, wastewater treatment plants have been documented as reservoirs of antibiotic resistant bacteria [48].

This study also observed that isotales from the hospital wastewater had a similar resistance pattern as those of the final effluent of the receiving WWTP as shown in Figure 4 and Figure 5 and Table 4 and Table 5 respectively. A high degree of similarity between the resistance patterns of the isolates recovered from hospital wastewater and final effluent of WWTP suggest that hospital wastewater could have contributed immensly to the resistances observed among the isolates from the final effluent. Hospitals are known to not only discharge pathogenic bacteria, most of which could be carrying resistance determinants into its wastewater, but also traces of antibiotics in urine, feces, as well as spilled and expired drugs that are improperly discarded into wash basins, are all channelled to the WWTP. Not all antibiotics in wastewater treatment plant undergo biodegradation. Many studies have shown low concentrations of antibiotics in treated water, which indicate a partial removal in waste water treatment plants [49,50,51,52]. Several studies have pointed out that WWTPs can provide favorable environmental conditions needed for antibiotic resistance genes transfer [2,53,54]. The possibility for potential spread of specific genes encoding antibiotic resistance in wastewater treatment plants by horizontal gene transfer has also been reported [55,56].

The presence of resistant enterococci in the hospital wastewater that is discharged to the Fort Hare WWTP and the observation of similaries in resistant patterns between hospital wastewater and final effluent of receiving WWTP suggest that the Victoria hospital wastewater could be a huge source of antibiotic resistant bacteria in the receiving WWTP. A similar study done by Lupo [57] about the relationship between antibiotic-resistance, bacterial community present in hospital wastewater, and wastewater treatment plant also identified hospital wastewater as a source of antibiotic resistance bacteria to the wastewater treatment plant. The limitations of this study are the sample size and low number of isolates analyzed. For a better picture to support these findings, a larger sample size and higher number of isolates needs to be analyzed. Further studies are therefore needed to validate these findings.

## 5. Conclusions

The results of the study revealed the challenges inthe chlorination process; hence, the final effluent discharged to the environment still contained multi-resistant enterococci. It also suggests that the hospital wastewater may contribute to the resistance enterococci in the final effluent of the WWTP as the resistance patterns of the isolates were very similar thus indicating that the former could most likely be a reservoir of antibiotic resistant bacteria. The findings call for interventions by relevant authorities as it has great public health implications for the health of humans and animals that are exposed to the receiving water body.

## References

[1] Periasamy D., Sundaram A. (2013). Environmental health A novel approach for pathogen reduction in wastewater treatment. J. Environ. Health Sci. Eng..

[2] Alam M.Z., Aqil F., Ahmad I., Ahmad S. (2013). Incidence and transferability of antibiotic resistance in the enteric bacteria isolated from hospital wastewater. Braz. J. Microbiol..

[3] Novo A., Manaia C.M. (2010). Factors influencing antibiotic resistance burden in municipal wastewater treatment plants. Appl. Microbiol. Biotechnol..

[4] Baquero F., Martinez J.L., Canton R. (2008). Antibiotics and antibiotic resistance in water environments. Curr. Opin. Biotechnol..

[5] Kemper N. (2008). Veterinary antibiotics in the aquatic and terrestrial environment. Ecol. Indic..

[6] Kümmerer K. (2009). The presence of pharmaceuticals in the environment due to human use—Present knowledge and future challenges. J. Environ. Manage..

[7] Byappanahalli M.N., Meredith B., Asja K., Zachery R.S., Valerie J.H. (2012). *Enterococci* in the environment. Microbiol. Mol. Biol. Rev..

[8] Kaltenthaler E.C., Pinfold J.V. (1995). Microbiological methods for assessing hand washing practice in hygiene behaviour studies. J. Trop. Med. Hyg..

[9] Ashbolt N.J., Grabow W.O.K., Snozzi M., Fewtrell L., Bartram J. (2001). Indicators of microbial water quality. Water Quality: Guidelines, Standards and Health.

[10] Fernandes S.C., Dhanashree B. (2013). Drug resistance & virulence determinants in clinical isolates of enterococcus species. Indian J. Med. Res..

[11] Jackson C.R., Fedorka-Cray P.J., Barrett J.B. (2004). Use of a genus- and species-specific multiplex PCR for identification of enterococci. J. Clin. Microbiol..

[12] Kafil H.S., Mobarez A.M., Moghadam M.F. (2012). Multidrug resistant and most virulent *Enterococcus faecium (strain 2653)*, isolated from hospitalized patient wound in Iran. Scholarly J. Med..

[13] Doğru A.K., Gençay Y.E., Ayaz N.D. (2010). Comparison of Virulence Gene Profiles of *Enterococcus* faecium and *Enterococcus* faecalis Chicken Neck Skin and Faeces Isolates。. Kafkas Univ Vet Fak Derg.

[14] Comerlato C.B., Resende M.C., Caierão J., d’Azevedo P.A. (2013). Presence of virulence factors in *Enterococcus faecalis* and *Enterococcus faecium* susceptible and resistant to vancomycin. Mem. Inst. Oswaldo. Cruz..

[15] Medeiros A.W., Pereira R.I., Oliveira D.V., Martins P.D., d’Azevedo P.A., van der Sand S., Frazzon J., Frazzon A.P.G. (2014). Molecular detection of virulence factors among food and clinical *Enterococcus faecalis* strains in south Brazil. Braz. J. Microbiol..

[16] Boyd D.A., Willey B.M., Fawcett D., Gillani N., Mulvey M.R. (2008). Molecular characterization of *Enterococcus faecalis N06-0364* with low-level vancomyc in resistance harboring a novel D-Ala-D-Ser gene cluster, vanL. Antimicrob. Agents Chemother..

[17] Courvalin P. (2006). Vancomycin resistance in gram-positive cocci. Clin. Infect. Dis..

[18] Lebreton F., Depardieu F., Bourdon N. (2011). D-Ala-D-SerVanN-type transferable vancomycin resistance in *Enterococcus faecium*. Antimicrob. Agents Chemother..

[19] Murray B.E., Nannini E.C., Mandell G.L., Bennet J.E., Dolin R. (2010). Glycopeptides (vancomycin and teicoplanin), streptogramins (quinupristin-dalfopristin), and lipopeptides (daptomycin). Principles and Practice of Infectious Diseases.

[20] Murray B.E. (1998). Diversity among multidrug-resistant enterococci. Emerg. Infect Dis..

[21] Teixeira L.M., Carvalho M.G.S.C., Facklam R.R., Murray P.R., Baron E.J., Jorgensen J.H., Landry M.L., Pfaller M.A. (2007). Enterococcus. Manual of Clinical Microbiology.

[22] Bai J., Paddock Z.D., Shi X., Li S., An B., Nagaraja T.G. (2012). Applicability of a multiplex PCR to detect the seven major shiga toxin—Producing *escherichia coli* based on genes that code for serogroup-specific o-antigens and major virulence factors in cattle feces. Foodborne Pathog. Dis..

[23] Ke D., Picard F.J., Martineau F., Menard C., Roy P.H., Ouellette M., Bergeron M.G. (1999). Development of a PCR assay for rapid detection of enterococci. J. Clin. Microbiol..

[24] Mannu L., Paba A., Daga E., Comunian R., Zanetti S., Duprè I., Sechi L. (2007). Comparison of the incidence of virulence determinants and antibiotic resistance between *Enterococcus faecium* strains of dairy, animal and clinical origin. Int. J. Food Microbiol..

[25] Vankerckhoven V., Autgaerden T.V., Vael C., Lammens C., Chapelle S., Rossi R., Jabes D., Goossens H. (2004). Development of a multiplex PCR for the detection of asa1, gelE, cylA, *esp*, and hyl genes in enterococci and survey for virulence determinants among European hospital isolates of *Enterococcus faecium*. J. Clin. Microbiol..

[26] Af Geijersstam A., Culak R., Molenaar L., Chattaway M., Røslie E., Peciuliene V., Haapasalo M., Shah H.N. (2007). Comparative analysis of virulence determinants and mass spectral profiles of Finnish and Lithuanian endodontic *Enterococcus faecalis* isolates. Oral Microbiol. Immunol..

[27] Manual on Antimicrobial Susceptibility Testing. http://www.asm.org/ccLibraryFiles/FILENAME/000000002484/Manual%20of%20Antimicrobial%20Susceptibility%20Testing.pdf.

[28] (2014). Performance Standards for Antimicrobial Susceptibility Testing; Twenty-Fourth Informational Supplement 2014.

[29] Nam S., Kim M.J., Park C., Park J.G., Lee G.C. (2012). Detection and genotyping of vancomycinresistant *Enterococcus* spp. by multiplex polymerase chain reaction in Korean aquatic environmental samples. Int. J. Hyg. Environ. Health.

[30] Okoh A.I. (2009). Impact of discharged wastewater final effluent on the physicochemical qualities of a receiving watershed in a suburban community of the Eastern Cape province. Clean Soil Air Water.

[31] Poh C.H., Oh H.M.L., Tan A.L. (2006). Epidemiology and clinical outcome of enterococcal bacteraemia in an acute care hospital. J. Infect..

[32] Kenzaka T., Takamura N., Kumabe A., Takeda K. (2013). A case of subacute infective endocarditis and blood access infection caused by *Enterococcus. durans.*. BMC Infect. Dis..

[33] Heim S., Lleo M.M., Bonato B., Guzman C.A., Canepari P. (2002). The viable but nonculturable state and starvation are different stress responses of *Enterococcus. faecalis*, as determined by proteome analysis. J. Bacteriol..

[34] Lleò M.M., Tafi M.C., Canepari P. (1998). Nonculturable *Enterococcus faecalis* cells are metabolically active and capable of resuming active growth. Syst. Appl. Microbiol..

[35] Lleò M.M., Bonato B., Benedetti D., Canepari P. (2005). Survival of *Enterococcal* species in aquatic environments. Fems. Microbiol. Ecol..

[36] Dupre I., Zanetti S., Schito A.M., Fadda G., Sechi L.A. (2005). Incidence of virulence determinants in clinical *Enterococcus faecium* and *Enterococcus faecalis* isolates collected in Sardinia (Italy). J. Med. Microbiol..

[37] Yousif N.M.K., Dawyndt P., Abriouel H., Wijaya A., Schillinger U., Vancanneyt M., Swings J., Dirar H.A., Holzapfel W.H., Franz C.M.A.P. (2005). Molecular characterization, technological properties and safety aspects of enterococci from Hussuwa, an African fermented sorghum product. J. Appl. Microbiol..

[38] Abriouel H., Ben Omar N., Molinos A.C., Lo’ pez R.L., Grande M.J., Martı’nez Viedma P., Ortega E., Can’amero M.M., Ga’lvez A. (2008). Comparative analysis of genetic diversity and incidence of virulence factors and antibiotic resistance among enterococcal populations from raw fruit and vegetable foods, water and soil, and clinical samples. Int. J. Food Microbiol..

[39] Kayaoglu G., Ørstavik D. (2004). Virulence factors of enterococcus faecalis: Relationship to endodontic disease. Crit. Rev. Oral. Biol. Med..

[40] Sedgley C.M., Molander A., Flannagan S.E., Nagel A.C., Appelbe O.K., Clewell D.B., Dahlén G. (2005). Virulence, phenotype and genotype characteristics of endodontic *Enterococcus spp*.. Oral Microbiol. Immunol..

[41] Reinthaler F.F., Posch J., Feierl G., Wüst G., Haas D., Ruckenbauer G., Mascher F., Marth E. (2006). Antibiotic resistance of *E. coli* in sewage and sludge. Water Res..

[42] Kümmerer K. (2004). Resistance in the environment. J. Antimicrob. Chemother..

[43] Martinez J.L. (2009). Environmental pollution by antibiotics and by antibiotic resistance determinants. Environ. Pollut..

[44] Czekalskiet N., Berthold T., Caucci S., Egli A., Bürgmann H. (2012). Increased levels of multiresistant bacteria and resistance genes after wastewater treatment and their dissemination into Lake Geneva, Switzerland. Front. Micro..

[45] Isogai N., Urushibara N., Kawaguchiya M., Ghosh S., Suzaki K., Watanabe N., Quiñones D., Kobayashi N. (2013). Characterization of *Enterococcus faecium* with macrolide resistance and reduced susceptibility to quinupristin/dalfopristin in a Japanese hospital: Detection of extensive diversity in erm(B)-regulator regions. Microb. Drug Resist..

[46] Moges F., Endris M., Belyhun Y., Worku W. (2014). Isolation and characterization of multiple drug resistance bacterial pathogens from waste water in hospital and non-hospital environments , northwest Ethiopia. BMC Res. Notes.

[47] Borhani B., Ahmadi A, Rahimi F., Pourshafie M.R., Talebi M. (2014). Determination of vancomycin resistant *Enterococcus. Faecium* diversity in tehran sewage using plasmid profile, biochemical fingerprinting and antibiotic resistance. Jundishapur. J. Microbiol..

[48] Da Silva M.F., Tiago I., Verissimo A., Boaventura R.A.R., Nunes O.C., Manaia C.M. (2006). Antibiotic resistance of *Enterococci* and related bacteria in an urban wastewater treatment plant. FEMS Microbiol. Ecol..

[49] Da Costa P.M.M., Vaz-Pires P.M., Bernardo F.M. (2006). Antimicrobial resistance in *Enterococci* spp. isolated from wastewater isolated in inflow, effluent and sludge from municipal sewage water treatment plants. Water Res..

[50] Zhou L., Ying G.G., Liu S., Zhao J.L., Yang B., Chen Z.F., Lai H.J. (2013). Occurrence and fate of eleven classes of antibiotics in two typical wastewater treatment plants in south China. Sci. Total Envirion..

[51] Akiyama T., Savin M.C. (2010). Populations of antibiotic-resistant coliform bacteria change rapidly in a wastewater *ef* fluent dominated stream. Sci. Total Envir..

[52] Gao L., Shi Y., Li W., Niu H., Liu J., Cai Y. (2010). Occurrence of antibiotics in eight sewage treatment plants in Beijing, China. Chemosphere.

[53] Gibs J., Heckathorn H.A., Meyer M.T., Klapinski F.R., Alebus M., Lippincott R.L. (2013). Occurrence and partitioning of antibiotic compounds found in the water column and bottom sediments from a stream receiving two wastewater treatment plant *ef* fluents in Northern New Jersey, 2008. Sci. Total. Envirion..

[54] Silva J., Castillo G., Callejas L., López H., Olmos J. (2006). Frequency of transferable multiple antibiotic resistance amongst coliform bacteria isolated from a treated sewage effluent in Antofagasta, Chile. Electron. J. Biotechnol.

[55] Sujatha S., Praharaj I. (2012). Glycopeptide resistance in gram-positive cocci: A review. Interdiscip. Perspect. Infect. Dis..

[56] Sahin I., Kaya D., Oksuz S., Okay A., Sencan I., Ozturk E. (2003). The frequency of extened spectrum beta lactamase and antibiotic susceptibility in clinical isolates of gram negative bacilli. Infeksiyon Dergisi.

[57] Lupo A., Coyne S., Berendonk T.U. (2012). Origin and evolution of antibiotic resistance: The common mechanisms of emergence and spread in water bodies. Front. Microbiol..

